# 2297: Dietary fat stimulates growth of pancreatic cancer growth through the cholecystokinin receptor

**DOI:** 10.1017/cts.2017.218

**Published:** 2018-05-10

**Authors:** Sandeep Nadella, Jill Smith, Julian Burks, Abdulhameed Al-Sabban, Juan Wang, Robin Tucker, Gloria Inyang

**Affiliations:** Georgetown - Howard Universities, Washington, DC, USA

## Abstract

OBJECTIVES/SPECIFIC AIMS: Epidemiologic studies have found that the incidence of pancreatic cancer is greatest in countries that consume diets high in fat. The gastrointestinal peptide cholecystokinin (CCK) is released from the duodenum in response to dietary fat. CCK has also been shown to stimulate growth of pancreatic cancer through the CCK receptor that is over-expressed on pancreatic cancer cells. The aim of this investigation was to determine if dietary fat promotes growth of pancreatic cancer through the actions of CCK at its receptor. METHODS/STUDY POPULATION: The effects of dietary fat on growth of murine Panc02 pancreatic cancer xenografts were studied in 3 different systems with immune competent mice: (1) pharmacologic blockade with a CCK receptor antagonist, (2) genetic knockout of the CCK receptor by CRISPR, and (3) in genetically engineered mice lacking the CCK peptide (CCK-KO). After injection of 2×106 Panc02 cells subcutaneously, mice were fed either a high-fat diet or a control diet for 37–42 days. Tumor volumes and weights were measured and histology performed. RESULTS/ANTICIPATED RESULTS: Dietary fat significantly increased the size of pancreatic cancer xenografts and this effect was reversed by CCK receptor blockade. Receptor antagonist therapy also significantly reduced tumor-associated fibrosis and increased the influx of CD8+ lymphocytes in the micro-environment. Panc02 cancer cells lacking CCK receptors failed to respond exogenous administration of CCK in vitro and to dietary fat in vivo. Dietary fat did not stimulate Panc02 tumor growth in CCK-KO mice. DISCUSSION/SIGNIFICANCE OF IMPACT: The mechanism by which dietary fat stimulates growth of pancreatic cancer is by CCK and this effect is independent of obesity. This is a significant finding because of the potential beneficial effects of medications which can block the effects of CCK in populations at risk for pancreatic cancer consuming a high-fat diet.

